# A cross-industry approach to the identification, prevention, and mitigation of workplace violence and mistreatment in the sign language interpreting field

**DOI:** 10.3389/fpubh.2026.1826495

**Published:** 2026-07-07

**Authors:** Gretchen Roman, Michael R. Privitera, Cristina Demian, Tanzy Love, Robert L. Weisman

**Affiliations:** 1 Department of Family Medicine, University of Rochester Medicine, Rochester, NY, United States; 2 Department of Psychiatry, University of Rochester Medicine, Rochester, NY, United States; 3 Department of Environmental Medicine and Public Health Sciences, University of Rochester Medicine, Rochester, NY, United States; 4 Department of Biostatistics and Computational Biology, University of Rochester Medicine, Rochester, NY, United States

**Keywords:** behavioral/interpersonal approaches, consumer/client-on-worker violence, horizontal violence, organizational/administrative exposure control strategies, safety and violence education (SAVE), sign language interpreting field, worker-on-worker violence, workplace violence and mistreatment

## Abstract

**Introduction:**

Both type II (consumer/client-on-worker) and type III (worker-on-worker) workplace violence and mistreatment exist within the sign language interpreting field, however type III (also known as horizontal violence or lateral aggression) is more commonly reported. Between 22 to 90% of sign language interpreters have witnessed and/or experienced forms of horizontal violence or behaviors associated with it and 0 to 8% have knowingly perpetrated horizontal violence against another interpreter. This perspective article aims to provide a cross-industry approach by sharing general practices and previous strategies used within the mental and general healthcare settings. We describe how these tools have been adapted to other industries and model how to apply them to the sign language interpreting field.

**Identification, prevention, and mitigation of workplace violence:**

Available regulatory guidance emphasizes employer responsibility for identifying workplace violence hazards and implementing evidence-informed prevention strategies. Safety and Violence Education (SAVE) was designed for front-line healthcare professionals who are exposed to multiple, well-documented risk factors for workplace violence. The original SAVE curriculum primarily emphasized the perceived predominance of consumer/client-on-worker violence in community mental health settings.

**Adaptation to the sign language interpreting field:**

Critical steps in adapting SAVE for sign language interpreters involved organizational/administrative exposure control strategies, like cultivating awareness of how organizational culture intersects with emotional and psychological safety and emphasized behavioral/interpersonal approaches by recognizing, preventing, and mitigating worker-on-worker aggression, bullying, and relational-based conflict.

**Discussion:**

The adapted SAVE sought to “break the cycle” of horizontal violence and establish a sustainable culture of safety, respect, and professional resilience among sign language interpreters.

## 1 Introduction

“Do we eat our young and one another?” That was part of a thesis title dating back to 2012, which reported on the experiences of workplace violence and mistreatment among sign language interpreters (1). In the quarterly magazine for the Registry of Interpreters for the Deaf (RID; the national professional association for sign language interpreters), leadership cited that horizontal violence, a type of workplace violence, was not new to the field but that it was becoming a more pressing concern because it continually presents in ways that “harm individuals and weaken our collective strength as a community of interpreters (2).”

**Figure 1:**
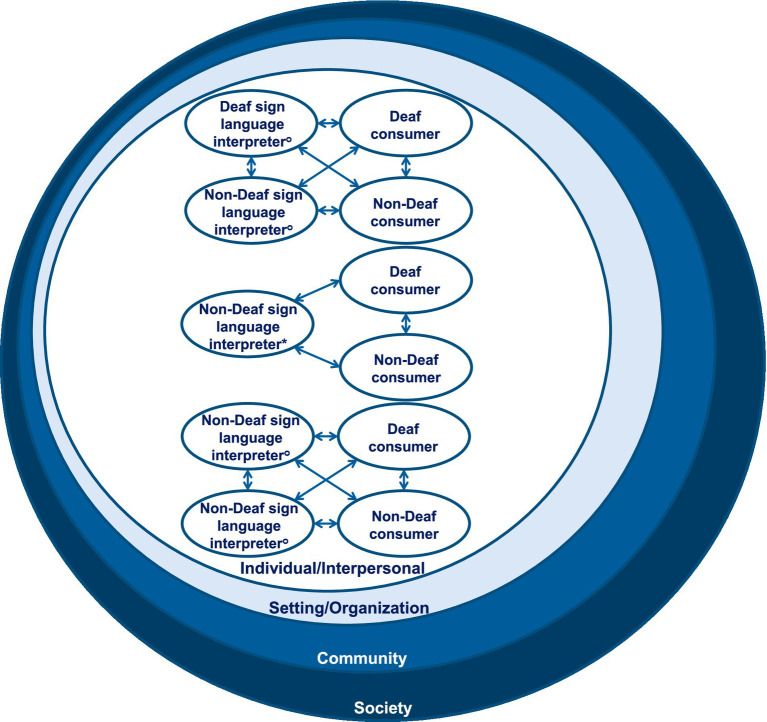
Socio-ecological view of the sign language interpreting field (*susceptible to type II violence; ^ᴑ^susceptible to BOTH type II and III violence).

Interpreters facilitate communication for individuals separated by a language barrier. Spoken language interpreters work between two spoken languages and rely on their hearing and speech to receive language and produce an interpretation. When compared with spoken language interpreters, sign language interpreters have unique cognitive, physical, and social demands (3, 4). Sign language interpreters may be Deaf or non-Deaf (hearing). Deaf interpreters work between visual languages and rely on their vision and gestural abilities to receive language and produce an interpretation, whereas non-Deaf interpreters work between language modalities (spoken language and sign language) and rely on their hearing, vision, speech, and gestural abilities. Different working conditions warrant that sign language interpreters work together as a team (Figure 1). Non-Deaf interpreters may work together if the interpreting assignment is longer than a set duration and Deaf and non-Deaf interpreters may work together when the non-Deaf interpreter may not be fluent in the visual language of the Deaf consumer (3–5).

Since the seminal research about workplace violence in the sign language interpreting field by Ott (2012), there have been increasing reports and attention drawn to its different types (6–16): criminal intent (type I), consumer/client-on-worker (type II), worker-on-worker (type III), and personal relationships (type IV). For consumer/client-on-worker violence, the perpetrator has a relationship with the business entity and becomes violent while receiving services. For worker-on-worker violence (also termed as horizontal violence or lateral aggression), the perpetrator is a worker or past worker of a business entity who threatens, physically, verbally, or emotionally attacks, or bullies another worker or past worker in the workplace (16).

Recent work examined the prevalence of both type II and type III violence in sign language interpreting. One report found 5% of sign language interpreters experienced sexual harassment by anyone while on the job, 13% were exposed to work-related physical violence, and 26% were subjected to work-related bullying (12). Specific to abusive behaviors experienced by sign language interpreters working in video relay (telecommunication between Deaf and non-Deaf parties), 91% of sign language interpreters had been admonished by a caller before being hung up on and 70% were involved in a phone call where a caller made sexual comments directed to them (13). Other work found 47% of sign language interpreters witnessed and/or experienced sabotage and 90% witnessed and/or experienced backstabbing, as behaviors associated with horizontal violence. The prevalence of other behaviors, such as non-verbal innuendo, covert or overt verbal affront, scapegoating, broken commitments and/or confidences, and microaggressions ranged somewhere in between (14). In another study, 22% of interpreters experienced another interpreter speaking common negative phrases to them and 71% experienced negative interpersonal interactions, as forms of horizontal violence. Other forms included acts of lateral aggression and actions negatively impacting the workplace or community, which ranged somewhere in between (11). Between 30 to 64% of interpreters witnessed these same forms of horizontal violence committed against another interpreter and between 0 and 8% knowingly perpetrated them (11).

Horizontal violence has been described as “infighting within a group of people who experience stress related to powerlessness (1).” Relating to systems of power, horizontal violence was witnessed and/or experienced between novice and experienced interpreters, students/mentees and educators/mentors, interpreters and interpreter referral agency representatives, Deaf and non-Deaf interpreter teams, and among Deaf interpreters (14). Between 19 to 57% of interpreters experienced another interpreter speaking common negative phrases to them, negative interpersonal interactions, acts of lateral aggression, and actions negatively impacting the workplace or community while they were interpreting students and between 27 to 54% of interpreters witnessed these same forms of horizontal violence committed against interpreting students (11). Deaf interpreters have expressed feeling stress from subjugated professional status when teaming with non-Deaf interpreters and indicated vertical violence, rather than horizontal violence, would be a more appropriate way to term their experience (15). Vertical violence occurs between colleagues who are presumed (Deaf and non-Deaf) to be in different hierarchical positions (17). Additionally, Deaf interpreters have acknowledged they tend to bully one another more than non-Deaf interpreters. This was attributed to their experience of oppression growing up and difficulty with deciphering how to parse their personal from professional experiences when working as an interpreter (15). While the majority of sign language interpreters reported “other” rationale without specifying or not knowing why horizontal violence was happening, some indicated that interpreters perpetrated such acts because they were too competitive, more seasoned with no patience, wanting to redirect attention to hide unethical decisions, accusatory of other interpreters stealing their work, jealous or had conflicting personalities, or were experiencing burnout from loss of agency (11). In addition to stress from subjugated professional status, professional hierarchies, and oppression, other causes of horizontal violence and emotionally or psychologically unsafe interpreting have been attributed to stress from constrained decision latitude or limited decision-making control and role stress (1, 8, 15).

At the recent biennial RID conference, preliminary data on the state of the sign language interpreting industry was reported (18). In this work, which entailed a 70-item survey with over 2,000 respondents and included 19 focus groups, horizontal violence experiences among interpreting practitioners ranged between 3 to 46% and included a workplace incident leading to legal action (3.1%), formal complaints (6.7%), altercations (11.5%), confrontations (32.9%), and criticism of work and skills (45.8%). This prompted comments from former and current RID leadership by way of the association’s quarterly magazine about addressing individual and cultural contributors to horizontal violence and turning challenges into opportunities (2, 19). Former leadership acknowledged that colleagues were courageous to begin naming the issue and called for training dedicated to providing “the language, strategies, and tools we need to navigate conflict and to cultivate healthier professional relationships (19).” Current leadership asked RID’s membership to (1) assume the best, rather than the worst in one another, (2) engage in dialog and questioning, rather than accusation or attack, (3) call out horizontal violence upon witnessing it, not to shame, but to interrupt harm, and finally, (4) engage with one another with compassion, guidance, and accountability to model a healthier way of working (2).

Workplace violence was one of main concerns impacting the mental health of sign language interpreters in our previous work (15). However, experience of workplace violence and mistreatment is not unique to sign language interpreters. It has been previously reported in other human service professions, like educational and mental and general healthcare settings (1, 14, 20–23). This perspective article aims to provide a cross-industry approach to the identification, prevention, and mitigation of types II and III violence common in the sign language interpreting field. We share general practices and previous strategies used within the mental and general healthcare settings. Then, we describe how these tools have been adapted to other industries and model how to apply them to sign language interpreting.

## 2 Identification, prevention, and mitigation of workplace violence

### 2.1 Risk factors, federal regulations, and high-risk industries

Risk factors that may contribute to the potential for workplace violence include exchanging money with the public, working alone or in isolated areas, providing services and care, working with volatile and unstable people, working where alcohol is served, working late at night, or in areas with high crime rates. There are no specific federal regulations in the United States that address workplace violence. Safety guidelines have been developed by the Occupational Safety and Health Administration (OSHA) and the National Institute for Occupational Safety and Health (NIOSH) for specific high-risk industries, like workers in healthcare (hospital caregivers, home care), social service, late-night retail, restaurants, and for taxi/ride share drivers; however, compliance is voluntary (24–27).

### 2.2 Exposure control strategies

Environmental, organizational/administrative, and behavioral/interpersonal exposure control strategies have been suggested by the Injury Prevention Research Center at the University of Iowa for the identification, prevention, and mitigation of workplace violence (16). Environmental approaches include engineering controls, such as adequate lighting, safe entrances and exits, defined cash handling procedures, and physical security enhancements. Organizational/administrative approaches entail developing programs, policies, and practices that aim to maintain a safe working environment. These may involve threat management procedures, like a team-oriented plan of action and timely management response to employee reports, as well as a “zero tolerance” and screening to identify potentially high-risk employees (16). Some suggest going beyond “zero tolerance” and argue that a shift in organizational culture is needed because workplace violence is not “principally a function of interpersonal conflict (28).” Behavioral/interpersonal approaches train employees on how to anticipate, recognize, and respond to conflict and potential workplace violence. Some examples include training to promote employee recognition of hazards and to facilitate appropriate employee response to incidents and comfort in reporting threatening behaviors (16).

### 2.3 A cross-industry approach

Front-line mental healthcare professionals routinely encounter both real and perceived threats to their personal safety, particularly when providing care to clients with complex psychosocial and behavioral needs. Safety and Violence Education (SAVE) was developed in the early 2000s and designed for front-line healthcare professionals in community-based settings who are exposed to multiple, well-documented risk factors for workplace violence (frequent client contact, unsupervised home and community outreach) and who work with individuals who may struggle with co-occurring substance use disorders and are experiencing acute distress and behavioral dysregulation (23).

SAVE is a modular training program (Table 1) created and delivered by the curriculum developer (RLW). Since its inception, SAVE has been conducted with several hundred mental health agencies across the United States and Canada. While program design, content, and related outcomes have been presented at international conferences, we recognize that regulatory oversight across high to low-income countries differs (29, 30). SAVE preparation begins with a pre-training assessment to tailor to the curriculum according to a particular audience’s needs and resources. Most training is delivered via onsite didactic seminars and typically, audiences have consisted of multidisciplinary groups of clinical providers, administrative staff, and agency leadership. In consideration of staff time, trainee needs and agency budgets, SAVE can be delivered in half- or full-day seminars. This approach allows for coverage of all relevant SAVE topics and interactive role-play exercises, rest breaks, and ample time for questions and answers. SAVE was augmented during the COVID-19 pandemic and now can also be delivered to audiences via web-based video conferencing. Additionally, it has been adapted to an interactive online learning format to promote access and provide refresher courses for learners (31).

**Table 1:** Content summary of safety and violence education (SAVE).

Sections	Topics	Subtopics/key elements
Violence risk and impact on staff	1. Introduction	Overview of training goals and objectives
	2. Need for Safety Training	Rationale for structured safety education
	3. What is WPV	Definitions and scope of workplace violence
	4. Impact of Violent Incidents	Effects on providers, clients, and others
	5. Risk Factors for Violence	Interpersonal factors; Institutional factors; Physical plant/environmental factors
	6. Prediction of Violence	Challenges and considerations in assessing potential violence
	7. Evaluation of Dangerousness	Practical tips for assessing risk and dangerousness
Violence prevention and mitigation strategies	8. Team Approach to Safe Practice	Interdisciplinary collaboration and shared responsibility
	9. Risk Reduction	Addressing systemic risks (e.g., short staffing)
	10. Assessing Each Situation	Situational Awareness Model
	11. Aggression Sequence	Recognition of escalation patterns
	12. Avoiding Common Mistakes	Frequent errors that increase risk
	13. Communication in Crisis Situations	Crisis communication strategies; Practical support techniques
	14. Safe Transport and Field Work	Home visit safety considerations
	15. Self-Defense	Hands-off approach principles
	16. High-Risk Situations	Identifying cases with increased risk potential
	17. Incident Review and Lessons Learned	Debriefing; Wellbeing; Leadership, mentorship, and supervision
	18. Q&A and Final Thoughts	Participant questions and training summary

WPV, workplace violence; Q&A, question and answer.

The original SAVE curriculum primarily emphasized the identification, prevention, and mitigation of type II workplace violence. This focus reflected the perceived predominance of client-related violence in community mental health settings and addressed direct physical or verbal assaults by clients or family members, exposure to vicarious trauma among staff, and the risk of retaliatory or reactive violence by staff toward clients under conditions of heightened stress or threat (32–34). Comparatively little attention was given to type III violence, such as lateral aggression, bullying, or intimidation among co-workers. As a result, interpersonal violence between staff members and organizational contributors to workplace aggression largely went unaddressed.

#### 2.3.1 Adaptation of SAVE to other industries

Building on the documented prevalence of workplace violence in healthcare settings, the demand for effective violence prevention and de-escalation training has increased substantially across the United States. Rising rates of violent incidents involving human service professionals beyond healthcare have underscored the need for training that is practical, affordable, and accessible. Regulatory bodies such as OSHA and NIOSH have consistently emphasized employer responsibility for identifying workplace violence hazards and implementing evidence-informed prevention strategies (24–27). Specifically, employers are responsible for providing a workplace that is “free from recognized hazards that are causing or are likely to cause death or serious physical harm (25).”

Due to sector-wide safety concerns, SAVE has been adapted for use across a broader range of healthcare and human service professionals that share similar environmental and interpersonal exposure risks. These adaptations also reflect recognition that type III workplace violence risk factors—such as frequent public contact, unpredictable environments, high emotional labor, and limited situational control—extend across multiple disciplines. As a result, SAVE has been implemented with diverse professional groups, including case management, emergency department staff, emergency medical services and other first responders, law enforcement, legal professionals, residential service providers, and sign language interpreters.

### 2.4 Suggestions for training delivery

Given the high rates of turnover and continual onboarding of new employees characteristic of contemporary workplaces, strategic implementation of workplace violence training is essential to ensure sustained workforce safety and competency. Effective implementation requires early introduction of comprehensive training as part of formal orientation processes. Ongoing competency maintenance should be supported through the provision of annual refresher training. Refresher courses should buttress learned skills while incorporating updates informed by emerging evidence and available regulatory guidance. Finally, continuous quality improvement processes are critical to the long-term effectiveness of workplace violence training. This includes systematic review of training outcomes, incorporation of interdisciplinary feedback, and alignment with other established occupational safety programs and industry standards. Cross-training and integration with broader organizational safety initiatives can further enhance program relevance, promote consistency across disciplines, and optimize delivery mechanisms for professionals working in complex and time-constrained environments.

## 3 Adaptation to the sign language interpreting field

In response to the former RID leadership’s call for dedicated training about horizontal violence, we model how to adapt general practices and previous strategies for sign language interpreters (19). As SAVE evolved beyond its original focus, the need to address horizontal violence became increasingly evident, particularly, within professions characterized by close collaboration, hierarchical structures, and constrained autonomy. Sign language interpreters may work in settings embedded within complex organizational cultures (Figure 1) that directly influence their psychosocial work environment, emotional well-being, and professional safety (35). Organizational culture has been shown to shape norms around communication, power, conflict resolution, and tolerance of incivility, all of which contribute to the emergence or mitigation of workplace violence (36, 37). Accordingly, a critical first step in adapting SAVE for sign language interpreters involved cultivating awareness of how organizational culture intersects with emotional and psychological safety for both individuals and teams.

Core elements of the SAVE curriculum (Table 1) were intentionally adapted to address the unique manifestations of type III violence experienced by sign language interpreters who participated in the Virtual Health Program for Sign Language Interpreters, a multi-component behavioral intervention delivered by the University of Rochester Medical Medicine. Workplace violence was one of eight occupational safety, health, and well-being concerns addressed. A pre-recorded didactic lecture (workplace violence video link in Supplemental materials) was viewed and question prompts were considered about the week’s topic in advance to joining a live hour-long virtual session. Facilitated by the same content expert featured in the pre-recorded video, the weekly sessions involved active learning as a large group and breakout sessions into smaller interpreting-setting specific affinity groups. Interpreting and captioning services were secured as appropriate to ensure accessibility for both Deaf and non-Deaf interpreters. While the original SAVE framework prioritized de-escalation of client aggression, these adaptations emphasized behavioral/interpersonal approaches by recognizing, preventing, and mitigating worker-on-worker aggression, bullying, and relational-based conflict. This shift also reflected growing evidence that both type II and III violence significantly contribute to moral distress, burnout, and workforce attrition (38, 39).

Following structured feedback from the Principal Investigator of the Virtual Health Program for Sign Language Interpreters (GR) and the evidence base on workplace violence in the sign language interpreting field (1, 2, 6–15, 18, 19), several targeted modifications were incorporated into the SAVE curriculum. A central adaptation involved reframing violence as a continuum ranging from “micro” to “mega” aggression (34). Within this framework, type III workplace violence was understood to most commonly manifest as microaggressions. These include, but are not limited to, incivility, disrespect, belittlement, toxic supervisory behaviors, and workplace bullying. Although often normalized or minimized within professional cultures, these behaviors have been shown to result in cumulative microtraumas that negatively affect psychological safety, professional identity, and job performance over time (8, 40, 41).

To enhance sign language interpreters’ capacity to recognize and respond to horizontal violence, the adapted SAVE further explored the common underlying contributors of stress related to powerlessness (1). The sign language interpreting field understands that such conditions align with the well-established occupational stress model of the Job Demand–Control Theory, which links limited autonomy and high demands to increased strain and interpersonal conflict (42–44). In tandem, the training emphasized the role of a sign language interpreter through a lens of both professionalism and humanism. Interpreters were encouraged to recognize the privilege and responsibility inherent to facilitating communication access. Preparation for high-pressure interactions was supported through a structured “5-P” performance management framework—Purpose, Plan, Preparation, Performance, and Progress—designed to reduce reactive behaviors and promote intentional, values-based professional conduct (45).

Leadership accountability and role-modeling were also integral organizational/administrative components of the adapted SAVE for sign language interpreters. Effective and trusted program leadership and veteran interpreters positioned as role models should be responsible for “setting the standard” of respectful, safe, and ethical practice and professional collaboration. This included explicit discussion of going beyond consistent enforcement of “zero tolerance” for repeated horizontal violence behaviors and fostering an organizational culture of emotional and psychological safety. Interpreters were additionally guided to acknowledge and actively avoid behaviors that contribute to undue stress, perpetuate oppressive dynamics, or normalize witnessed incivility.

Finally, the adapted SAVE addressed the risk of professional burnout, emphasizing the downstream consequences of chronic exposure to horizontal violence, including emotional exhaustion, depersonalization, and reduced professional efficacy (46). By confronting inherent power differentials within diverse interpreter teams and between interpreters and leadership, SAVE promotes proactive, team-based strategies that foster trust, collaboration, and shared accountability. The adapted SAVE sought to “break the cycle” of type III workplace violence and establish a sustainable culture of safety, respect, and professional resilience among sign language interpreters.

## 4 Discussion

The goal of this perspective was to provide a cross-industry approach to the identification, prevention, and mitigation of workplace violence and mistreatment in the sign language interpreting field. Most of evidence that supported our conceptual discussion and practical recommendations came from Master theses and the available literature about workplace violence from professions in the health sector. High quality empirical evidence, specifically evaluating interventions for workplace violence among sign language interpreters, is limited. While formal outcome evaluation of the Virtual Health Program for Sign Language Interpreters efficacy is forthcoming, it examined the impact of a multi-component behavioral intervention on sign language interpreter well-being, rather than workplace violence alone. The original SAVE curriculum has a history of widespread delivery and has been received by many different groups; however, the adapted SAVE for sign language interpreters has, to date, been applied in this one instance. Preliminary implementation suggests potential outcomes, including improved recognition of horizontal violence, enhanced emotional and psychological safety, and increased confidence in addressing interpersonal conflict. These outcomes are hypothetical at this stage and warrant further systematic evaluation. Future research should examine the effectiveness of this adapted training through mixed-methods approaches that assess incidence of reported type III violence, interpreter burnout, job satisfaction, and staff retention over time. Longitudinal studies may be particularly valuable in determining whether early exposure to SAVE during interpreter onboarding produces sustained improvements in professional behavior and organizational culture (16). Additionally, future research should explore how shifts in organizational culture, like leadership accountability, role-modeling, and power-sharing practices interact with training outcomes, as well as whether SAVE can be further refined to address intersectional factors such as race, gender, disability, and professional marginalization within interpreter teams. Once empirical evaluation of the adapted SAVE for sign language interpreters has been conducted, further research should focus on comparative effective studies. These studies can assess the impact of SAVE relative to alternative workplace violence interventions, helping to inform best practices for workplace violence among interpreters. Together, these efforts would contribute to the development of evidence-informed, sector-specific violence prevention strategies and strengthen the broader literature on workplace safety in human service professions.

## Data Availability

The original contributions presented in the study are included in the article/Supplementary material, further inquiries can be directed to the corresponding author.
