# Supplementary material for: A cross-industry approach to the identification, prevention, and mitigation of workplace violence and mistreatment in the sign language interpreting field
Source: Front Public Health. 2026 Jul 7;14:1826495. doi: 10.3389/fpubh.2026.1826495 (PMC13384915; doi:10.3389/fpubh.2026.1826495)
Supplement: Supplementary file 1 [file Supplementary_file_1.pdf]

Supplemental materials

Access to the pre-recorded video about workplace violence that was used in the Virtual Health Program for Sign Language Interpreters can be found: https://rochester.hosted.panopto.com/Panopto/Pages/Viewer.aspx?id=cfcfaccd-74d7-436f-9226-b2e501209dc7
